# Preparation and structural characterization of chitosan‑sodium alginate nanocapsules and their effects on the stability and antioxidant activity of blueberry anthocyanins

**DOI:** 10.1016/j.fochx.2024.101744

**Published:** 2024-08-15

**Authors:** Boyu Chen, Chao Ai, Yuanju He, Yimei Zheng, Lei Chen, Hui Teng

**Affiliations:** College of Food Science and Technology, Guangdong Ocean University, Guangdong Provincial Key Laboratory of Aquatic Product Processing and Safety, Guangdong Province Engineering Laboratory for Marine Biological Products, Guangdong Provincial Engineering Technology Research Center of Seafood, Key Laboratory of Advanced Processing of Aquatic Product of Guangdong Higher Education Institution, Zhanjiang 524088, China.

**Keywords:** Anthocyanins, Chitosan, Nanocapsules, Stability, Sodium alginate

## Abstract

This study prepared a nanocapsule (NPs) from chitosan (CS) and sodium alginate (ALG) and used them to enhance the stability of blueberry anthocyanins (BA) The optimal NPs was obtained at pH value of 3.25, BA concentration of 0.5 mg/mL and mixing ratio of CS to ALG of 1:1 (*W*/W). Further, the formation of composite NPs was confirmed by a series of characterization methods. The CS-BA-ALG NPs appeared spherical, smooth, and evenly distributed when observed under an optical microscope and transmission Electron Microscope. The X-ray Diffractometer and Fourier Transform Infrared spectroscopy data proved that electrostatic interaction and hydrogen bonding are dominant forces to form NPs. Thermogravimetric analysis and differential scanning calorimetry results demonstrated that the CS-BA-ALG NPs system significantly improved the thermal stability of anthocyanins. In addition, it was also proved that CS-BA-ALG NPs showed high antioxidant capacity and protection capacity.

## Highlights

• Chitosan and sodium alginate formed nanocapsules to load anthocyanins.
• The characteristics of nanocapsules have small particle size, good stability and high encapsulation rate
• The formation of nanocapsules is mainly through electrostatic interaction and hydrogen bonding
• Degradation of anthocyanins was inhibited at harsh conditions after encapsulation.

## 1 Introduction

Anthocyanins are a group of natural pigments that are widely found in fruits vegetables and cereals. Among these, blueberries, known as the “king of anthocyanins”, are an important source of these pigments (Xie, Lu, Zhou, Hao, & Chen, 2022). Blueberry anthocyanins (BA) have been found and verified to have various physiological functions, such as antioxidant activity (Jiao et al., 2018), anti-inflammatory (Ma, Du, Li, Zhu, & o. M. S., 2021), bactericidal effects, and so on. However, BA have poor stability under natural conditions and are easily decomposed by light, oxygen, temperature, and other factors (Enaru, Drețcanu, Pop, Stǎnilǎ, & Diaconeasa, 2021).

To address this issue and further enhance the value of BA, various chemical modification or physical embedding methods can be used to modify their structure and improve their stability. Currently, the commonly used methods include structural modification (Wang, Liu, Zhuang, & Fei, 2022), complexation with metals (Tachibana, Kimura, & Ohno, 2014), and self-assembly coating technology (Yu, Yu, Dong, & Xia, 2022). Structure modification of anthocyanins can be achieved through chemical or enzymatic acylation, which involves reducing hydrophilic group attack and increasing steric hindrance. However, the cost is high. Complexation with the metals usually requires the addition of other stabilizers, such as chondroitin sulfate, which may raise safety concerns (Tang, He, & Fan, 2023). Self-assembly coating technology can effectively enhance the stability of anthocyanins through the use of colloidal systems. This technology allows for the incorporation of anthocyanins into different food systems and dietary supplements to maintain food color (as food additives) (Cheng et al., 2023). Additionally, this technology can achieve controlled intestinal release or even direct gastrointestinal absorption, thereby increasing the concentration of bioactive compounds in the target tissue and achieving more advanced functions (Jiang & Zhang, 2023). Formulations based on biopolymers derived from organic materials, such as polysaccharides, proteins, and liposomes, offer high safety and biocompatibility, which can help improve the stability and accessibility of anthocyanins (Chen & Hu, 2020).

Chitosan (CS) is a natural cationic polysaccharide and has been widely used as a biopolymer for nano-carrier preparation. Protonation of the amino group at acidic pH is a unique advantage of CS, which can easily interact with negatively charged biopolymers (Elgadir et al., 2015). Sodium Alginate (ALG) is a water-soluble polyanionic polymer, and it's non-toxic, biodegradable, low-cost and easy to obtain (Paques, van der Linden, van Rijn, & Sagis, 2014). CS and ALG can form high stability and good compatibility nanoparticles by non-covalent bonding such as ionic and hydrogen bonds, which can be used as a new carrier to contain anthocyanins.

Therefore, a self-assembling nanocapsules (CS-BA-ALG NPs) was prepared and optimized to improve the stability of BA, and the effect of the NPs on the functional activity of BA was evaluated. The study investigated the effects of pH, BA concentration, the proportion of CS and ALG, and total concentration on the particle size, zeta potential, and encapsulation efficiency (EE) was investigated. The physical characteristics of the NPs were analyzed using optical microscopy, transmission electron microscopy (TEM), X-ray diffractometer (XRD), and Fourier infrared spectrometer (FTIR). Additionally, the antioxidant activity and stability of CS-BA-ALG NPs were assessed. The encapsulation system is expected to provide a relevant research basis for BA homeostasis, which may help provide new ideas and scientific basis for the development of naturally sourced anthocyanins-relevant products in pharmaceutical and food industry applications.

## 2 Materials and methods

### 2.1 Chemical reagents

CS (deacylation degree ≥85) of food grade was purchased from Shandong Weikang Biomedical Technology Co., LTD. (Linyi, Shandong, China). BA (25% anthocyanin purity) was purchased from Xi ‘an Shengqing Biotechnology Co., LTD. (Xi ‘an Shaanxi, China). ALG (purity ≥90) of food grade was ordered from Qingdao Mingyue Seaweed Group Co., LTD. (Qingdao, Shandong, China).

### 2.2 Preparation of anthocyanins samples

The anthocyanins solution was prepared according to the procedure of (Mao et al., 2023) with some modifications. The concentration of 2 mg/mL blueberry anthocyanins solution (BAS) was prepared by weighing 0.2 g of blueberry powder and dissolved in 100 mL ultra-pure water, stirred under a dark environment at room temperature for half an hour to make it fully dissolved, stored under 4 °C and used within 3 days.

### 2.3 Preparation of NPs

The NPs were prepared according to (Yu et al., 2022) with some modifications.

Preparation of CS-ALG NPs: 1% CS and ALG working solutions were prepared by fully dissolving their respective powders in 1% (*v*/v) acetic acid and ultra-pure water, respectively. Subsequently, using HCl (0.1 mol/L) and NaOH (0.1 mol/L) solutions to adjust the pH to 2.75, 3, 3.25, 3.5, 3.75 and 4. The ALG solution was then added drop by drop to the CS solution and stirred at 700 rpm (SHL-2 A, Jiangsu, China) for 2 h at room temperature. The CS-ALG NPs suspension was prepared using a high-intensity ultrasonic processor equipped with a probe (diameter 10 mm, frequency 20 kHz, maximum power 1000 W) for 8 min (power 600 W, 5 s on and 5 s off). During the ultrasound process, the sample was placed in darkness and ice at room temperature.

Preparation of CS-BA-ALG NPs: The ALG solution was added to a configured BAS of 2 mg/mL to make a final concentration of anthocyanin concentration between 0.2 and 1.0 mg/mL. Then the ALG-BA solution was prepared by stirring at a constant speed of 700 rpm for 30 min at room temperature. Under the same conditions, the ALG-BA solution was added drop by drop to the CS solution, and the CS-BA-ALG NPs suspension was prepared by ultrasonic treatment. The effects of different factors and their concentrations on the NPs size and stability were inspected by single-factor experiments. Factors and their concentration ranges proceeded as follows: total concentrations of CS and ALG (1–2 mg/mL), the ratio between them (5:1–1:4), pH (2.75–4) and BA concentration (0.2–1 mg/mL) on particle size and zeta potential of NPs.

### 2.4 LC-MS/MS analysis for BA

The method was modified based on (Zhang et al., 2024). The LC-MS/MS analysis was conducted using the Waters XEVO G2-XS Q-Tof ultra-performance liquid chromatography system coupled to the time-of-flight mass spectrometry (Waters, Massachusetts, USA). Liquid chromatographic separations were performed using a Waters ACQUITY UPLC BEH C18 column (2.1 × 100 mm, 1.7 μm). The mobile phase consisted of 0.1% formic acid in water (solvent A) and methanol (solvent B). The temperature-controlled column oven was set at 35 °C, while the sample manager temperature was maintained at room temperature (23 °C). The flow rate was set at 0.3 mL/min, and the injection volume was 1 μL. The total running time for the analysis was 15 min. The samples were eluted using a linear solvent gradient as follows: 10% B, 0–2 min; 10–60% B, 2–10 min; 60–10% B, 10–12 min; 10% B, 12–15 min;

For mass spectrometry, data was acquired in Media Source Extensions (MSE) mode using negative electrospray ionization (ESI) under sensitivity mode. The acquisition range was set from 100 to 1000 *m*/*z*. The capillary voltage was set at 2 kV, while the sampling cone voltage was set at 40 V. The source offset was set at 80 V, and the source temperature was maintained at 120 °C. The desolvation temperature was set at 450 °C with a cone gas flow of 50 L/h and a desolvation gas flow of 700 L/h. The data was processed using MassLynx V 4.1 software.

### 2.5 Measurement of particle size and zeta potential

The method was modified based on (Song et al., 2024). The composite CS-BA-ALG NPs were characterized by the ZETASIZER NANOZSE (Guangdong Shengze Technology Co., LTD., Dongguan, Guangdong Province). The test temperature was maintained at 25 °C. Dynamic light scattering (DLS) technology was employed to obtain particle size and zeta potential data at a scattering angle of 90° and a refractive index of 1.46.

### 2.6 EE and loading capacity (LC) of BA in NPs

The content of BA was determined using the Folin-Ciocalteu method (Yu et al., 2022). The CS-BA-ALG NPs suspension was separated by an ultra-high-speed cryogenic centrifuge (Eppendorf Ltd., Hamburg, Germany) at 10000 rpm for 20 min to collect the supernatant. The amount of BA in the NPs was calculated by subtracting the amount of BA not embedded in the supernatant from the total amount of BA added to the CS-BA-ALG NPs. The absorbance of the solution was measured at 760 nm using the Varioskan fully automatic enzyme labeler.

The EE and LC of BA in NPs was calculated according to the following eq. (1) and (2) and a standard curve was drawn:

(1) $$\mathrm{EE}\;\left(\%\right)=\frac{\mathrm{TC}-\mathrm{FC}}{\mathrm{TC}}\times 100\%$$

(2) $$\mathrm{LC}\;\left(\%\right)=\frac{\mathrm{TC}-\mathrm{FC}}{\mathrm{MW}}\times 100\%$$

where TC— The total amount of BA added in supernatant (mg/mL);

FC — Amount of free BA (mg/mL).

MW — The total weight of CS and ALG (mg).

### 2.7 Morphological analysis of NPs system

First, the camera port of the optical microscope (Olympus CKX53) was used to capture images. A suspension of 10 μL CS-ALG NPs and CS-BA-ALG NPs was added to a slide, and a cover glass was placed over it for observation under the optical microscope (eyepiece×objective lens of 10 × 40). The specimen was positioned directly facing the center of the light hole.

The morphology of the NPs was further observed using TEM. The CS-ALG NPs suspension and CS-BA-ALG NPs suspension were diluted with ultra-pure water at a volume ratio of 1:10. A 10 μL diluted sample was then placed on a copper net and stained with 2% phosphotungstic acid for 1 min. After drying at 25 °C, morphological analysis of the samples was performed by transmission electron microscopy (JEM-2100JEOL, Showa, Tokyo, Japan) at an accelerated voltage of 120 kV (Yu et al., 2022).

### 2.8 FTIR

This method refers to (Dong et al., 2023) someone and makes some modifications. The FTIR spectra of CS powder, ALG powder, BA powder, CS-ALG NPs, and CS-BA-ALG NPs were obtained using a Fourier Transform infrared spectrometer (TENSOR 27, Bruker, Germany) with a maximum resolution of 0.5 cm^−1^. The purpose of this analysis was to identify and analyze the presence or changes in functional groups or chemical bonds within the samples. The sample sheets were scanned within an infrared range of 400 to 4000 cm^−1^.

### 2.9 XRD

The crystal structure of the sample was determined by a D/MAX2500 X-ray diffractometer (Rigaku, Japan) with a continuous mode, and 2θ Angle was set from 5° to 70° with a scanning rate of 2°/min (Dong et al., 2023). The total area and amorphous regions area of XRD pattern was calculated by Origin, and the crystallinity calculated as follows:

(3) $$\text{Crystallinity}\;\left(\%\right)=\left[\left(A-B\right)/A\right]\times 100\%$$

where A was the total area; B was the amorphous regions area.

### 2.10 TG and DSC

A relative method (Dong et al., 2023) was used for the TG and DSC analysis with a slight modification. The freeze-dried samples (5–10 mg) were weighed and transferred onto a synchronous thermal analyzer (STA 449F3, Jupiter, Netzsch Scientific Instruments Trading Co., Germany). The scan range was set at 45–440 °C, with a temperature rise rate of 10 °C/min, and a flow of dry nitrogen at 20 mL/min.

### 2.11 Determination of antioxidant capacity

#### 2.11.1 DPPH free radical scavenging assay

The determination of DPPH free radical scavenging activity was modified based on the method recorded by (Zheng et al., 2023).

Different samples were mixed with DPPH-ethanol solution (0.0039 g DPPH was completely dissolved in anhydrous ethanol, diluted to 100 mL, and stirred away from light for 2 h) at a ratio of 1:4. After 30 min in darkness, the absorbance of the mixed solution was measured at 517 nm using a Varioskan Flash Spectra (Thermo Fisher Scientific Co., Ltd., USA). The DPPH free radical scavenging activity of anhydrous ethanol was used as a control and calculated using the following eq. (4):

(4) $$\text{DPPH scavenging activity}\;\left(\%\right)=\left(1-\frac{A\mathrm{i}-\mathrm{Aj}}{A_{0}}\right)\times 100\%$$

where, A_i_ — sample absorbance;

A_j_ — absorbance of blank sample;

A_0_— The absorbance of the control.

#### 2.11.2 ABTS^+^ free radical scavenging assay

The ABTS^+^ free radical scavenging activity was determined according to the method of (Zheng, Liu, Yu, Farag, & Shao, 2023) with a slight modification. The ABTS^+^ original solution was prepared by mixing ABTS^+^ with potassium persulfate solution at a ratio of 1:1. The original ABTS solution was placed in a dark place for 12 h to get a balance. The original ABTS^+^ solution was diluted with ultra-pure water until the absorption value was 0.7 ± 0.1. The sample was then mixed with a diluted ABTS^+^ solution in a ratio of 1:19 and incubated for 6 min before reading its absorbance at 734 nm under Varioskan Flash Spectra (Thermo Fisher Scientific Co. Ltd., USA). The ABTS^+^ scavenging activity was calculated using the following eq. (5):

(5) $${\text{ABTS}}^{+}\;\text{scavenging activity}\;\left(\%\right)=\left(1-\frac{A\mathrm{i}-\mathrm{Aj}}{A_{0}}\right)\times 100\%$$

where, A_i_ — sample absorbance;

A_j_ — absorbance of blank sample;

A_0_ — The absorbance of the control.

### 2.12 Stability analysis

The stability of the NPs was analyzed in different treatments. The effects of light and temperature on the storage stability of CS-BA-ALG NPs were also compared with BA. The stability analysis was according to (Xie et al., 2023) with some modifications.

#### 2.12.1 Light stability

The CS-BA-ALG NPs and the BA solution of the same volume (4 mL) were added separately into the test tube. They were then irradiated in front of a UV lamp at a distance of 10 cm for varied periods (1 h, 2 h, 3 h, 4 h, 6 h). The retention rate (RR) for BA was calculated using the pH differential method. The formula was as follows by (Zhao et al., 2020).

(6) $$\mathrm{RR}\left(\%\right)=\frac{\mathrm{Current}\;\mathrm{BA}\;\mathrm{content}}{\mathrm{Total}\;\mathrm{BA}\;\mathrm{content}}\times 100\%$$

#### 2.12.2 Thermal stability

BA (4 mL) and CS-BA-ALG NPs were added to test tubes, and the samples were then heated in a water bath at 90 °C for different times (2 h, 4 h, 6 h, 8 h, 10 h), and then rapidly cooled in ice to reach room temperature. The RR calculation method for BA was employed as described above.

### 2.13 Statistical analysis

This statistical analysis method was designed with reference to (El-Hadary, Sulieman, El-Shorbagy, & Control, 2023). The tests were exhausted triplicate in line and therefore the data were analyzed using the means, variance by Microsoft Office Excel (2016), paired sample *t*-test, and one-way ANOVA variance analysis by IBM SPSS version 25.0 software (SPSS Inc., Chicago, IL, USA) at the extent of probability of (*p* < 0.05), and Origin (Version 8.0; Origin Lab, USA) were used for further data processing and charting.

## 3 Results and discussion

### 3.1 Identification of blueberry anthocyanins (BA)

**Fig. 1:**
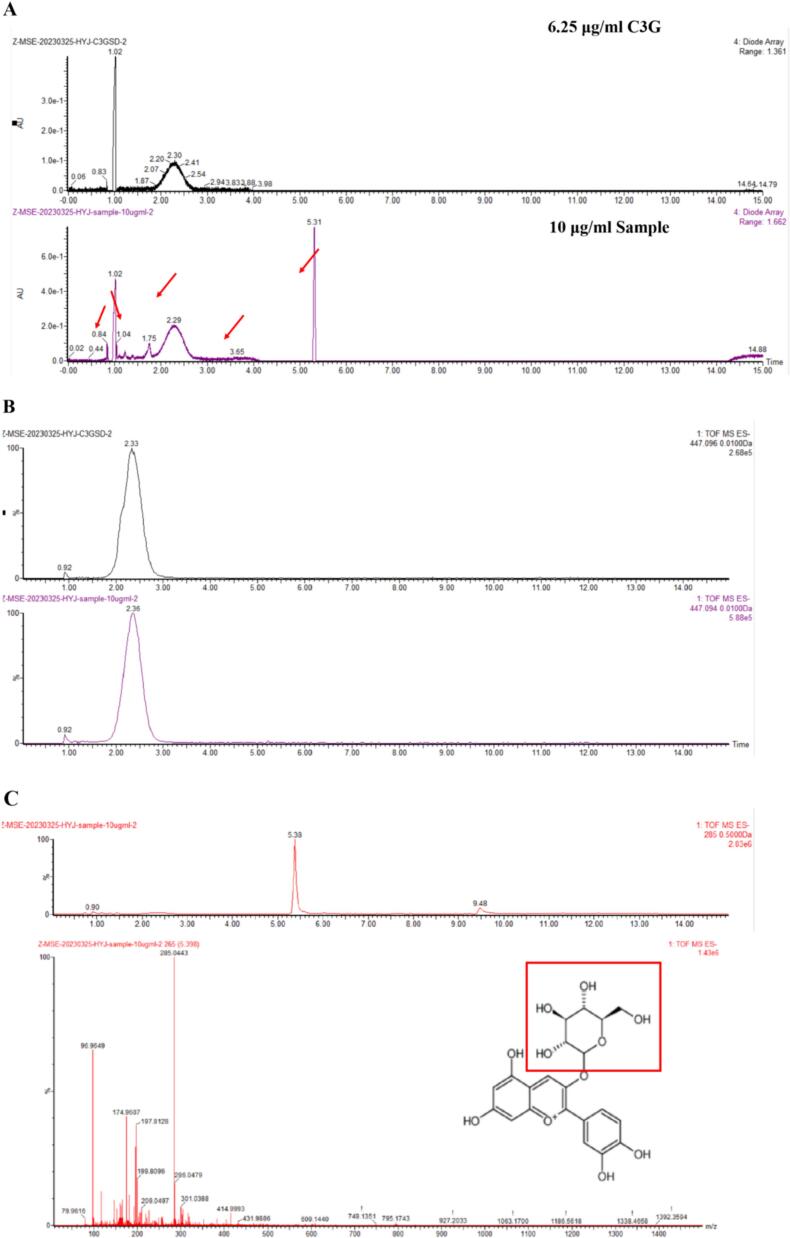
Qualitative analysis of blueberry anthocyanins. (A) Petunidin-3-pyrannoside. (B) Mass spectrometry analysis of blueberry anthocyanins.

Fig. 1A and B show the HPLC-DAD profile of BA. The main anthocyanins in blueberry was identified as cyanidin-3-O-glucoside, as confirmed by HPLC-DAD and LC-MS/MS.

### 3.2 Effect of preparation factors on the particle size, zeta potential (ZP), and EE of nanocapsules (NPs)

In order to optimize the NPs system, a single-factor experimental design was used to inspect the effects of different factors and concentrations on the particle size, zeta potential (ZP), and EE.

**Fig. 2:**
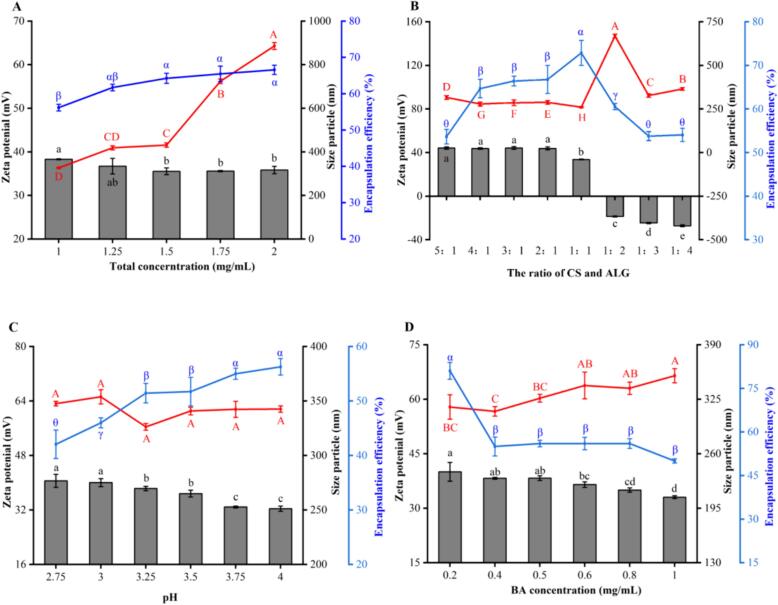
Effect of total concentration of chitosan (CS) and sodium alginate (ALG) (A), the ratio of CS and ALG (B), pH (C), and blueberry anthocyanins (BA) concentration (D) on particle size, Zeta potential and Encapsulation efficiency of CS-BA-ALG NPs (CS/ALG: 1:1 (*W*/W), BA concentration: 0.5 mg/mL, CS and ALG total concentration: 1 mg/mL, BA concentration: 0.5 mg/mL).

Fig. 2A shows that the size of NPs tends to increase significantly with the increase of the total concentration of CS and BA. When the concentration of polymers is low, the addition of ALG to CS may form a smaller condensation nucleus If the concentration of the polymer is raised, a larger condensation nucleus may be formed (Saboktakin, Tabatabaie, & R.A., Ramazanov, M.A., 2010). (Zhang et al., 2022) also suggested that the complexation process should be performed in dilute polymer solutions with concentrations lower than the overlap concentration of polymer chains, in order to provide uniform complexation conditions as much as possible. It also can avoid partial high-concentration effects in the mixing step. When the total concentration is 1 mg/mL, the EE of BA is 56.17%. The EE increased gradually with the increase of total concentration. The total concentration of polymers higher, the electrostatic interaction sites increase more, resulting in an increase in the number of NPs, which makes it easier to embed BA. However, when the total concentration is 1.25 mg/mL, the incremental change of EE is relatively small, and the EE have no significance after it (*p* > 0.05). In conclusion, the total concentration was 1 mg/mL which has a high embedding rate and loading rate (Appendix 1 A), so our study selected the option of the total concentration is 1 mg/mL to proceed the later experiment.

The particle size was the smallest when the CS-ALG ratio (*w*/w) was 1:1 from Fig. 2B (*p* < 0.05), which may be caused by more interaction between ALG and CS at a high level. The result was consistent with the research results reported by (Tan, Xie, Zhang, Cai, & Xia, 2016). The increase in particle size may be have two reasons, one is the accumulation of residual polysaccharide, which causes the NPs size to increase significantly, the other may be due to CS molecular weight is greater than ALG. The entanglement of CS chains will lead to more complexation and condensation (Saboktakin et al., 2010). Looking at the overall, NPs with ZP values >30 ± 5 mV are able to maintain a stable dispersion state for a longer period of time (Kim, Kim, Lee, & Lee, 2021). The figure show that the ZP value got 18.67 mV (lower than 20 mV) when the CS-ALG ratio (*w*/w) was 1:2, which might condense or flocculate due to weak reaction, resulting in a significant increase in particle size (*p* < 0.05). Since CS and ALG is a polycationic polysaccharide and polyanionic polysaccharide, the ZP value of NPs increases depends on the change of the CS-ALG ratio. The encapsulation efficiency of BA was the highest when the CS-ALG ratio (w/w) was 1:1. The high encapsulation efficiency of NPs may be due to both of CS and ALG are hydrophilicity and high molecular weight which help to form a denser structure as a barrier with a number of holes, thus effectively trapping BA.

Fig. 2C illustrates the effect of pH on the stability of CS-BA-ALG NPs. The figure shows that the smallest particle size was observed at pH 3.25. Although the change is not significant (*p >* 0.05), the particle size has an increasing trend whether the pH is higher or lower than pH = 3.25. The phenomenon may be due to the decreased ionization degree and solubility of CS at a high pH value. Conversely, at low pH values, the ionization degree of the carboxyl group on the molecular chain of ALG is not conducive to the formation of uniform particles in the system (Tan et al., 2016). The electrostatically driven complexation between polyelectrolytes of ionizing groups, such as amino, carboxyl, and sulfuric acid groups, is strongly dependent on the pH value of the medium. The reduction of group ionization leads to the shrinkage of particle size and the reduction of ZP (Rahaiee, Shojaosadati, Hashemi, Moini, & Razavi, 2015). (Feng et al., 2020) found that with a continuous increase in pH value, the dissociation degree of sodium alginate increased, leading to an increase in negatively charged carboxyl ions. Therefore, the maximum degree of CS and ALG complex to form particles in property pH. In addition, all the NPs with ZP values are 30–40 mV so that they can have a good storage stability. Additionally, the encapsulation efficiency of BA significantly increased with an increase in the pH (*p* < 0.05). As pH increases, the dissociation of the carboxyl group in ALG increases, increasing in negatively charged COO-ions. After mixing with CS solution, the dissociated COO-ion rapidly interacts with NH_3_^+^, through electrostatic gravitational binding, and BA to be encapsulated in NPs (Feng et al., 2020).

The concentration of anthocyanins also affects the NPs. Fig. 2D exhibited that the particle size of the NPs generally increased, and the ZP gradually decreased when the BA concentration increased from 0.2 mg/mL to 1.0 mg/mL (*p* < 0.05). The ZP of NPs is related to its surface charge density. The increase in anthocyanins concentration causes the phenolic hydroxyl group in the structure to react with the amino group in chitosan, resulting in a decrease in positive charge on the surface of the particles and a decrease in zeta potential values (Cai et al., 2021)**.** As illustrated in Fig. 2D, the EE of BA decreased with an increase in the BA concentration (p < 0.05). (Zhou et al., 2020) found that as the ratio of polyphenols to polysaccharides increased, the encapsulation efficiency of NPs reduced from 54.24% to 46.26%. Therefore, when the concentration of 0.5 mg/mL BA is selected, smaller particle size and higher EE can be obtained.

According to the above analysis, when the total concentration of CS-ALG is 1 mg/mL, the ratio (*w*/w) is 1:1, the pH is 3.25, and 0.5 mg/mL BA is selected, the particle density of CS-BA-ALG NPs is the highest, the particle diameter is the smallest, and the EE and LC are the best.

### 3.3 FTIR analysis

**Fig. 3:**
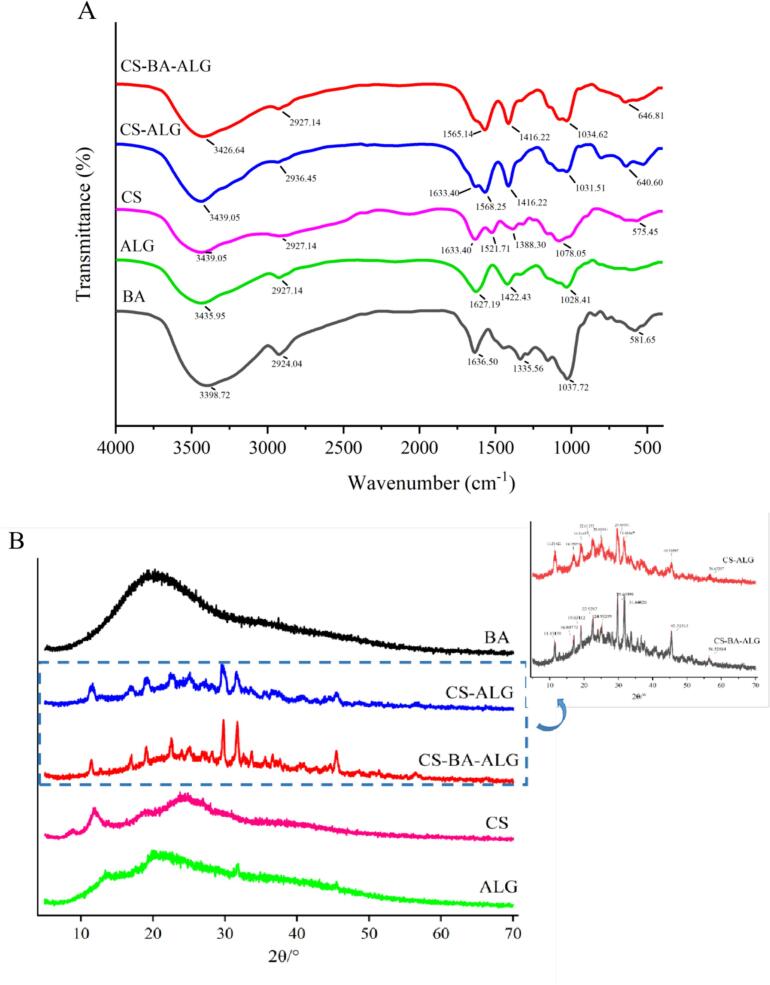
The FTIR spectrum (A) and the X-ray diffraction (B) of blueberry anthocyanins (BA), sodium alginate (ALG), chitosan (CS), CS-ALG complex, CS-BA-ALG complex.

FTIR was utilized to analyze the potential interaction between anthocyanins, chitosan, and alginate using infrared spectroscopy (Fig. 3A). The figure show that there are mainly six absorption peaks. The absorption band at 3398.72 cm^−1^ indicates the formation of hydrogen bonds between the phenol hydroxyl groups of anthocyanins (Biao et al., 2018). The absorption bands at 2924.04 cm^−1^ and 1636.50 cm^−1^ are characteristic functional groups of polyflavonoids (Bridson, Al-Hakkak, Steward, & Al-Hakkak, 2019), and the characteristic peak of flavonoid tannins is near 1037.72 cm^−1^ (Pereira Jr., Queiroz de Arruda, & Stefani, 2015).

CS exhibited a faint absorption band at 2927.14 cm^−1^, which corresponded to the antisymmetric stretching vibration of the methyl group. Other characteristic absorption peaks of the CS were also detected, including the C=O stretching vibration absorption peak at 1633.40 cm^−1^ amide I, the N—H bending vibration absorption peak at 1521.71 cm^−1^, and the CH_2_ and CH_3_ bending vibration absorption peak at 1388.30 cm^−1^. The absorption peak at 1078.05 cm^−1^ represented the asymmetric stretching vibration of the C-O-C bond of the glycoside bond. Similar absorption bands have been declared in other studies as well (Bai & Wu, 2022).

Owing to the carboxyl anion (COO-), the spectrum of ALG has two absorption bands at 1627.19 cm^−1^ and 1422.43 cm^−1^ (Hernandez-Nava, Lopez-Malo, Palou, Ramirez-Corona, & Teresa Jimenez-Munguia, 2020). There was another absorption band at 2927.14 cm^−1^, which was slightly stronger than CS. And absorption band at 1028.41 cm^−1^ was confirmed as the stretching vibration of the absorption peak of the C-O-C bond of the glycosidic bond.

In CS-ALG NPs, the N—H stretching peak of the amide (1633.40 cm^−1^) in CS and the absorption band of the carboxyl anion (1568.25 cm^−1^) in ALG disappeared. The peak at 1416.22 cm^−1^ has stronger intensity than CS (1388.80 cm^−1^). Compare with CS and ALG, the peak has blue or red shift. Both the results indicated that the -NH_3_^+^ in the CS was reacted with O—H and -COO groups of ALG resulted in the bending vibration of amide bond -NH and the stretching vibration of C=O (Ding, Mo, Zhang, Bi, & Kong, 2021). Similar results also appeared at 640.60 cm^−1^, a new peak appeared.

Compared with CS-ALG NPs, the O—H absorption peak of CS-BA-ALG NPs showed a blue-shift phenomenon, and the O—H absorption peak changed from 3439.05 cm^−1^ to 3426.64 cm^−1^. The formation of hydrogen bonds dispersed the density of electron clouds, thereby reducing the stretching vibration frequency, which indicated non-covalent interaction among BA, CS, and ALG (Needham, 2013). At the same time, the peak at 1633.40 cm^−1^ shifted to 1565.14 cm^−1^, corresponding to the C=C bond vibration on the aromatic ring skeleton, which is in agreement with the aromatic compounds contained in the anthocyanin extract. This phenomenon indicates an interaction between the hydroxyl group in BA and the amide group of CS-ALG NPs (Javid, Dounighi, & Arbabi Bidgoli, 2024). It can also be observed that the absorption peak of BA near 1335.36 cm^−1^ disappeared, which also represented the embedding of BA in CS and ALG.

The above results showed that the amide bond formed by carboxyl group and amino group, and the electrostatic interaction between them promoted the preparation of CS-ALG and CS-BA-ALG NPs, and the driving force of BA coating on the NPs came from the hydrogen bond formed between polar groups.

### 3.4 XRD analysis

Fig. 3B shows the X-ray diffraction of free BA, ALG, CS, CS-ALG NPs, and CS-BA-ALG NPs. The BA had a chubby wide peak when 2θ = 10–30°, which indicated that it had an indeterminate structure. The CS possessed four diffraction peaks at 2θ = 8.64°, 11.86°, 18.92° and 24.32°, respectively. Among them, the diffraction peak at 2θ = 11.86° was a semi-wide peak, because of the hydrated crystal structure caused by water molecules in the lattice, and the rest were wide peaks, indicating that chitosan was mainly an amorphous structure. (S Moalla et al., 2021) also found that the CS exhibited diffraction peaks at 2θ = 8.24°, 11.26°, 16.15°, 17.94° and 23.65°, and when ALG is cross-linked with CS, eight characteristic peaks appear, 2θ = 11.51°, 16.98°, 19.21°, 22.61°, 25.05°, 29.69°, 31.68°, 45.59° and 56.62°. By then, a large number of hydrogen bonds are generated due to the interaction between ALG and CS, forming an ordered structure, and more intense characteristic peaks are produced. After loading the BA onto the NPs, it was observed that the displacement changed as compared with CS-ALG, which means that the addition of BA might lead to changes in the structure of CS-ALG (Ding et al., 2021). At the same time, the CS-ALG NPs crystallinity was 21.41% and the CS-BA-ALG NPs crystallinity was 22.64% (Table 1). BA addition increased the crystallinity of NPs. It might be explained by the fact that the incorporation of BA inhibited the interaction between CS and ALG, which then improved the number of molecular fragments, thus promoting the crystallization process (Zheng, Liu, et al., 2023). (Liu et al., 2023) also reported similar results. Therefore, due to the interaction of CS, ALG, and BA, CS-BA-ALG NPs formed an ordered and highly crystallized structure, which was able to improve the stability of BA.

**Table 1:** The crystallinity of the CS-ALG, and CS-BA-ALG NPs.

Sample name	Crystallinity (%)
**CS-ALG**	21.41
**CS-BA-ALG**	22.64

### 3.5 Microscope analysis of NPs

The surface microstructures of CS-ALG NPs and CS-BA-ALG NPs were observed by TEM and binocular optical microscope.

**Fig. 4:**
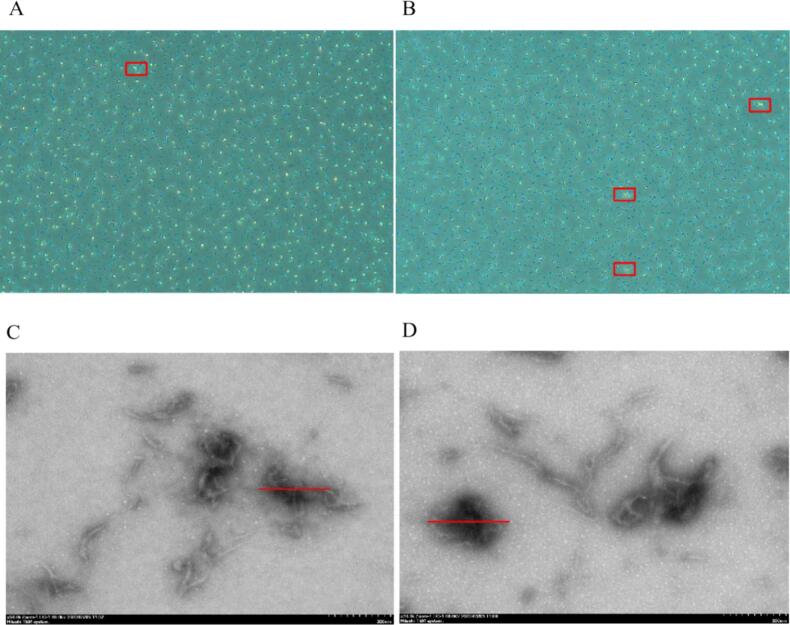
The micrograph (A-B) of chitosan-alginate (CS-ALG) complex and chitosan-blueberry anthocyanins-alginate (CS-BA-ALG) complex; The TEM (C—D) of CS-ALG complex and CS-BA-ALG complex.

Micrographs in Fig. 4A and Fig. 4B show that both of the distribution of NPs is uniform, and CS-BA-ALG NPs has more relative aggregation than CS-ALG NPs. It might suggest larger particle size of CS-BA-ALG NPs.

TEM results confirmed that the uncoated CS-ALG NPs had an irregular shape with a particle size of about 220 nm (Fig. 4C). While the coated CS-BA-ALG NPs showed a relatively uniform spherical structure with a particle size about 250 nm (Fig. 4D). (Tang, He, & Fan, 2023) pointed out that the strong electrostatic interaction between hydroxypropyl chitosan (HPCS) and sodium alginate (ALG) led to the irregular and large structural morphology of HPCS-ALG composite gel. In addition, after the addition of purple corn bract anthocyanin (PCBA), the HPCS-ALG/PCBA composite nanogel showed a relatively uniform spherical structure. In the presence of anthocyanins, non-covalent interaction between polysaccharides and anthocyanins arises the formation of hydrogen bonds, leading to shape deformation and agglomeration of NPs (Yu et al., 2022). The NPs of CS-BA-ALG observed in dynamic light scattering (DLS) was 320 nm, while the particle diameter observed by TEM was small, which might be explained by the presence of NPs in the form of air-dried particles (Jeevitha & Amarnath, 2013). The increase in particle size may be due to the insertion of BA into CS-ALG NPs. In addition, anthocyanin-coated NPs have been found to have a darker color in the core, indicating a higher electron density distribution in this region (Zhang & Zhao, 2015), which can also suggest that the BA was embedded in NPs.

### 3.6 Antioxidant capacity of NPs

**Fig. 5:**
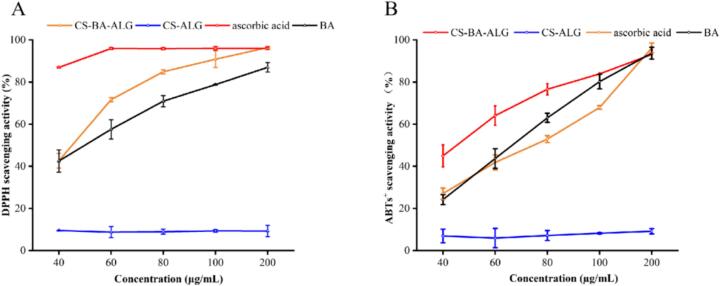
In vitro antioxidant activities of ascorbic acid, blueberry anthocyanins (BA), chitosan-blueberry anthocyanins-alginate (CS-BA-ALG) NPs and CS-ALG NPs. (A) DPPH radical scavenging assay; (B) ABTS^+^ radical scavenging assay; CS-BA-ALG NPs were based on the actual concentration of BA encapsulated in the NPs (calculated from LC%).

DPPH is a stable free radical that is widely used to evaluate the antioxidant activity of natural products and synthetic compounds. As illustrated in Fig. 5A, anthocyanins were effective in scavenging DPPH free radicals via providing hydrogen atoms and transferring electrons through rich phenol hydroxyl groups, and their scavenging ability was linearly correlated with their concentrations. It is worth noting that at the same concentration, the DPPH scavenging activity of CS-BA-ALG NPs is 10–20% higher than that of free BA. The enhancement of antioxidant activity of the BA in CS-ALG NPs may be due to the protective effect of encapsulation, which prevents the BA from being affected by environmental conditions and other factors. In contrast, CS-ALG NPs nearly showed no DPPH scavenging activity and did not change with increasing concentrations. This limitation may be due to the fact that CS and ALG do not have particularly obvious antioxidant groups on the materials themselves, or it may be related to the electrostatic interaction between CS and ALG, which combines to mask the groups in CS and ALG that react with DPPH molecules (Aljawish et al., 2014).

As shown in Fig. 5B, similar to the DPPH scavenging activity discussed above, the ABTS^+^ scavenging activity of CS-BA-ALG NPs increased with the increase of sample concentration, and within a certain range, the ABTS^+^ antioxidant activity of CS-BA-ALG NPs is higher than BA.

### 3.7 TGA and DSC analysis

TGA and DSC measurements were employed to analyze the differences in thermal properties of free BA, CS-ALG NPs before and after loading with BA.

**Fig. 6:**
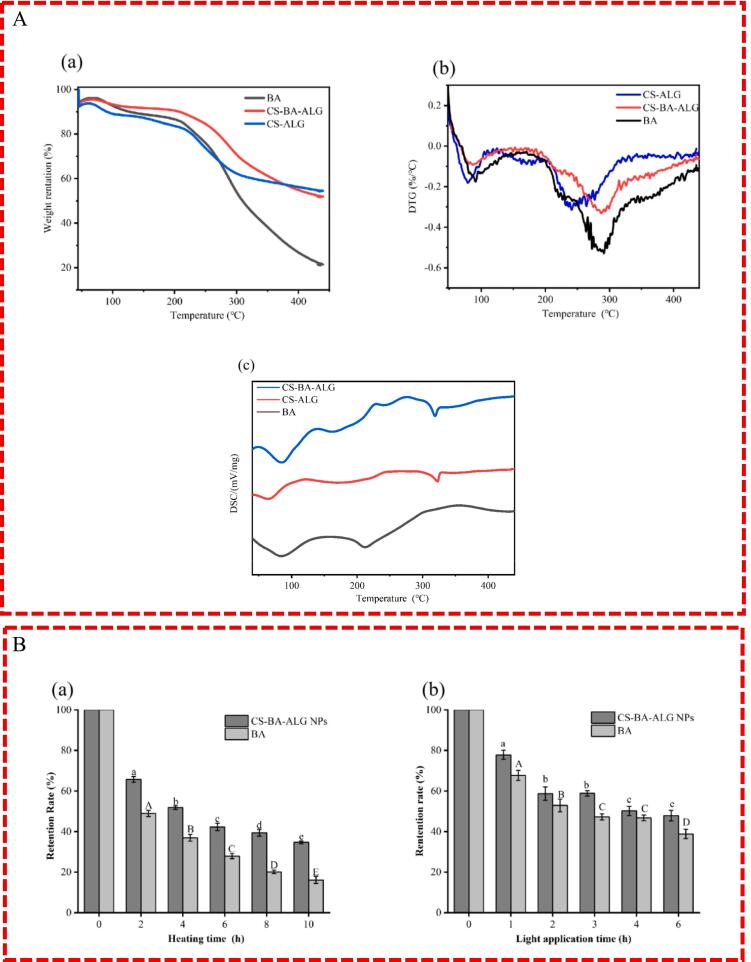
Thermal properties analysis for blueberry anthocyanins (BA), chitosan-blueberry anthocyanins-alginate (CS-BA-ALG) NPs and CS-ALG NPs (A-a) Thermogravimetric analysis, (A-b) Derivative Thermogravimetry and (A-c) Differential scanning calorimetric; Effect of different treatment time at 90 °C on storage stability of CS-BA-ALG and free BA (B-a) and effect of different time under ultraviolet lamp irradiation storage stability of CS-BA-ALG and free BA (B-b).

TGA and derivative thermogravimetric (DTG) for the BA, CS-ALG NPs, and CS-BA-ALG NPs are shown in Figs. 6A-a and 6A-b. As we know, at the same temperature, the smaller the weight loss of the sample, the higher the thermal stability of the sample. Near 65–80 °C, it has similar endothermic properties, and its weight loss is due to the desorption of bound water and small molecular water components on the sample. Compared with free BA, the loss of CS-BA-ALG nanocapsules was smaller, while the water loss temperature of CS-BA-ALG nanocapsules increased as compared with CS-ALG. It indicated that the interaction between BA, CS and ALG turned the structure more compact and the water retention got better, which was consistent with the results reported by (Dong et al., 2023). The mass of BA began to degrade at 180 °C and reached the maximum weight loss rate at 270 °C, which was maintained until the end of the reaction (about 440 °C). The thermal degradation process mainly consisted of two stages, including the evaporation of physical and chemical-bound water and degradation into phenolic acids and aldehydes (Wang, Ma, Zhang, & Zhao, 2014). At the same time, the thermal degradation of CS-BA-ALG went through three stages. In addition to the water loss in the first stage, in the second stage (temperature range 180–240 °C), CS-BA-ALG nanocapsules showed good hydrophilicity, and the slight change in mass change rate was related to the water loss of the hydrophilic groups of the particles (Ahmady et al., 2023). The third stage degradation temperature was around 250–340 °C and the maximum weight loss rate reached near 290 °C, which was relative to the structural decomposition of CS and ALG (Dong et al., 2023). Compared with free BA, the mass loss rate and the maximum weight loss rate of CS-BA-ALG NPs greatly reduced, which indicated that the anthocyanins embedded in the nano-system had good thermal stability. The thermal degradation of CS-ALG NPs was similar to that of CS-BA-ALG NPs, but the melting point is lower and each stage mass loss is greater than CS-BA-ALG NPs.

DSC is able to confirm the formation and thermal degradation of complexes because the dissociation and thermal degradation of complexes during heating lead to endothermic peaks. The DSC analysis results of pure BA, CS-ALG NPs and CS-BA-ALG NPs are given in Fig. 6A-c. We found that the three samples had an endothermic peak near 65–80 °C, and the microstructure of CS-ALG NPs changed after the addition of BA, leading to an increase in melting temperature. Nanocapsules showed a small exothermic peak near 230–250 °C probably because the degradation and depolymerization of polyelectrolyte, and the comparable data was revealed by **(**Kunjumon, Viswanathan, & Baby, 2021) as well. The CS-ALG NPs and CS-BA-ALG NPs had an obvious endothermic peak at 320–330 °C, indicating the interaction effect among CS, ALG, and BA. The addition of BA resulted in a slight decrease in the melting temperature of CS-BA-ALG NPs. A possible explanation for this phenomenon might be due to the hydrogen bond between chitosan and anthocyanins changed original regular structure (Wang et al., 2023). The BA had a significant absorption peak at 200–210 °C, which was not found in the DSC maps of NPs, suggesting that the BA was successfully embedded in the NP system.

### 3.8 Stability analysis

During the storage process, the BA is easily degraded into colorless substances owing to its instability. The C4 hydroxyl ring of BA tends to convert to chalcone and produce intermediate products in the presence of light and oxygen. The intermediates are further oxidized over time to some lysates, such as 2, 4, 6-trihydroxybenzaldehyde, which results in discoloration and degradation, and the degradation rate increases with the increase of temperature (Zhao et al., 2020). Therefore, the stability of anthocyanins is greatly affected by light and temperature.

As can be observed from Fig. 6B-a, both BA and CS-BA-ALG NPs show a decreasing trend with the increase of heating time at 95 °C. However, compared with free BA, the CS-BA-ALG NPs has a high thermal stability and a relatively minor loss of anthocyanins (*p* < 0.05). This experiment was similar to the results reported by (Kim, Jeong, Baek, & Lee, 2023), who confirmed the embedding of nanoscale systems can improve the stability. As confirmed by the previous differential scanning calorimetry thermal and thermogravimetric analysis (Fig. 6A), we confirmed that the electrostatic interaction between CS and ALG in the prepared NPs was able to protect the core material of BA, and reduce the effect of temperature.

Similar to the trend of thermal stability, the results of Fig. 6B-b exhibited that anthocyanins contents of both BA and CS-BA-ALG had a decreasing trend with the increase of illumination time under ultraviolet light (*p* < 0.05), but the degradation mechanism mainly came from the excitation of flavonoid cations (Zang et al., 2021). (Wang, Liu, et al., 2022) also found that nanocarriers could improve the stability of curcumin under ultraviolet light. Therefore, the CS-ALG NPs encapsulates BA, which can improve its stability in the environment.

## 4 Conclusion

In this study, CS-BA-ALG NPs were successfully prepared. The optimal process in this study was a pH value of 3.25, BA concentration of 0.5 mg/mL, CS and ALG ratio of 1:1(*W*/W), and total concentration of 1 mg/mL. The NPs has small particle size, high encapsulation efficiency and load capacity, and good stability. It has a good application value in the development of nutritional health products or functional foods.

## CRediT authorship contribution statement

**Boyu Chen:** Writing – original draft, Methodology, Investigation, Data curation, Conceptualization. **Chao Ai:** Writing – review & editing, Supervision. **Yuanju He:** Validation, Investigation. **Yimei Zheng:** Validation, Investigation. **Lei Chen:** Writing – review & editing, Supervision, Project administration, Funding acquisition. **Hui Teng:** Supervision, Project administration, Funding acquisition, Conceptualization.

## Declaration of competing interest

The authors declare that they have no known competing financial interests or personal relationships that could have appeared to influence the work reported in this paper.

## Data Availability

The data that has been used is confidential.

## Appendix A Appendix 1

Effect of total concentration of chitosan (CS) and sodium alginate (ALG) (A), the ratio of CS and ALG (B), pH (C), and blueberry anthocyanins (BA) concentration (D) on Load capacity of CS-BA-ALG NPs. (CS/ALG: 1:1 (W/W), CS and ALG total concentration: 1 mg/mL, pH: 3.25, BA concentration:0.5 mg/mL).
